# A New Framework for Assessing Equid Welfare: A Case Study of Working Equids in Nepalese Brick Kilns

**DOI:** 10.3390/ani10061074

**Published:** 2020-06-22

**Authors:** Stuart L. Norris, Laura M. Kubasiewicz, Tamlin L. Watson, Holly A. Little, Atish K. Yadav, Sajana Thapa, Zoe Raw, Faith A. Burden

**Affiliations:** 1 The Donkey Sanctuary, Sidmouth, Devon EX10 0NU, UK; laura.kubasiewicz@thedonkeysanctuary.org.uk (L.M.K.); tamlin.watson@thedonkeysanctuary.org.uk (T.L.W.); holly.little@thedonkeysanctuary.org.uk (H.A.L.); zoe.raw@thedonkeysanctuary.org.uk (Z.R.); faith.burden@thedonkeysanctuary.org.uk (F.A.B.); 2 Animal Nepal, Dhobhighat, Lalitpur 44600, Nepal; animalnepalatish@gmail.com (A.K.Y.); animalnepalsajana@gmail.com (S.T.)

**Keywords:** welfare aggregation, equid welfare, brick kilns, resource allocation, handler attitude

## Abstract

**Simple Summary:**

Brick kilns are difficult environments in which to maintain a high level of equid welfare, with equids experiencing poor nutrition, inadequate veterinary care, wounds and musculoskeletal problems from ill-fitting equipment used to transport heavy loads—all of which are exacerbated by hot and dusty working conditions. The Equid Assessment, Research and Scoping (EARS) tool was used to understand the health, behaviour, nutrition, housing and working conditions of working equids in Nepalese brick kilns to better understand ways of improving their welfare. The information gathered using the EARS tool was summarised using the Welfare Aggregation and Guidance (WAG) tool to pinpoint areas of welfare concern and suggest possible mitigation strategies. Overall, results indicate that to improve the welfare of equids working in Nepalese brick kilns, there should be better access to clean water, both when working and stabled, which would improve nutritional welfare. Equipment should be removed during rest periods, which may reduce the number of scars and swellings observed. There should be improvements to the housing regime to allow the equids to rest and recuperate. We show that the attitudes of handlers towards their equid has an impact on the welfare conditions of the equid and suggest training programs to address this, specifically focusing on the impacts of using harmful practices such as hobbling or tethering.

**Abstract:**

Equids fulfil many different roles within communities. In low- to middle-income countries (LMICs), in addition to providing a source of income, equids also provide essential transport of food, water, and goods to resource-limited and/or isolated communities that might otherwise lack access. The aim of this investigation was to understand the welfare conditions that donkeys, mules, and horses are exposed to whilst working in Nepalese brick kilns. To understand the welfare conditions of equids in Nepalese brick kilns, the Welfare Aggregation and Guidance (WAG) tool in conjunction with the Equid Assessment, Research and Scoping (EARS) tool was used to understand the health, behaviour, nutrition, living and working conditions in brick kilns. Further analysis of individual EARS responses focused on key indicator questions relating to demographic information was used to investigate specific areas of welfare concern and attitudes of handlers towards their equids. Trained staff carried out welfare assessments between December 2018 and April 2019. The information gathered using the EARS tool was summarised using the WAG tool to pinpoint areas of welfare concern and suggest possible strategies to mitigate poor welfare conditions and suggest areas to improve the welfare of equids. Overall, the results indicate that to improve the welfare of equids working in Nepalese brick kilns, there should be better provision of clean water, both when working and stabled, equipment should be removed and shade provided during rest periods, with improvements made to housing to allow the equids to rest and recuperate when not working. Further work should also focus on collaborating with owners and equid handlers to improve their attitudes and practices towards their equids. Such improvements can be implemented via training of equid handlers and kiln owners whilst using the EARS and WAG tools to provide a sound basis on which to monitor the effectiveness and impact of education programs on equid welfare.

## 1. Introduction

Working equids support humans in many ways, including carting goods and people [1], carrying packs to transport bricks and construction materials [2,3], for draught power in agriculture [4,5], carrying water, in rubbish collection [6], and for ceremonial use [4,7,8]. A wide spectrum of welfare issues can be encountered throughout the world depending on differences in environment [9], levels of urbanisation [10], and the type of work equids conduct [3,5,11,12]. Associated welfare concerns are equally varied, with equids reported to experience poor body condition [12] and abnormal hoof shape in agricultural settings in Guatemala [13], malnutrition and harness-related wounds in the markets of Ethiopia [14] and high levels of respiratory disease and cases of tethering in draft and pack animals in Pakistan [15] and Egypt [16]. Whilst an array of welfare issues are known to exist, research into the prevalence of these problems within large populations and their relationship to demographic status remains sparse [11,12,13].

In recent decades, there has been an increased interest in monitoring animal welfare [17,18,19]. Whilst assessments for equids have been developed [8,11,20,21], none provided easily comparable assessments that cover all situations equids may be subject to [11,13,22], with the exception of the recently developed Equid Assessment, Research and Scoping (EARS) tool [23]. The EARS tool provides a rapid assessment approach to collect information relating to the welfare of equids via a set of context-specific protocols. The EARS assessments draw on both animal- and resource-based questions to build a comprehensive understanding of equid welfare [11,23,24]. The Welfare Aggregation and Guidance (WAG) tool builds on the EARS tool by providing a rapid, repeatable and transparent method for grading equid welfare [15]. By aggregating the most indicative measures of welfare from the EARS assessments at the population level, the WAG process results in separate grades for five welfare categories: housing conditions, working conditions, health, nutrition and behaviour. The WAG tool overcomes problems associated with other welfare aggregation approaches by using a single aggregation method [15]. The WAG process identifies groups of equids with the poorest levels of welfare and provides a standardised system to aid decision making when allocating resources and monitor the efficacy of interventions to improve equid welfare [15].

Brick kilns have been found to be particularly difficult environments in which to maintain a high level of equid welfare [3,12,16], with equids experiencing poor nutrition [12,25], inadequate veterinary care, wounds and musculoskeletal problems from ill-fitting equipment used to pull heavy loads, which is exacerbated by generally hot and dusty working conditions [16,26,27]. Here, we use responses to EARS questions in conjunction with aggregated welfare grades from the WAG tool to outline the welfare conditions of equids in the brick kilns of Nepal. We examine differences in welfare between different demographic groups, work types and regions. Next, we investigate the drivers of poor welfare within each of the WAG categories. Finally, we explore the attitude of equid handlers in Nepalese brick kilns and how this links to each welfare category, in order to make practical recommendations for improving equid welfare.

## 2. Materials and Methods

**Figure 1:**
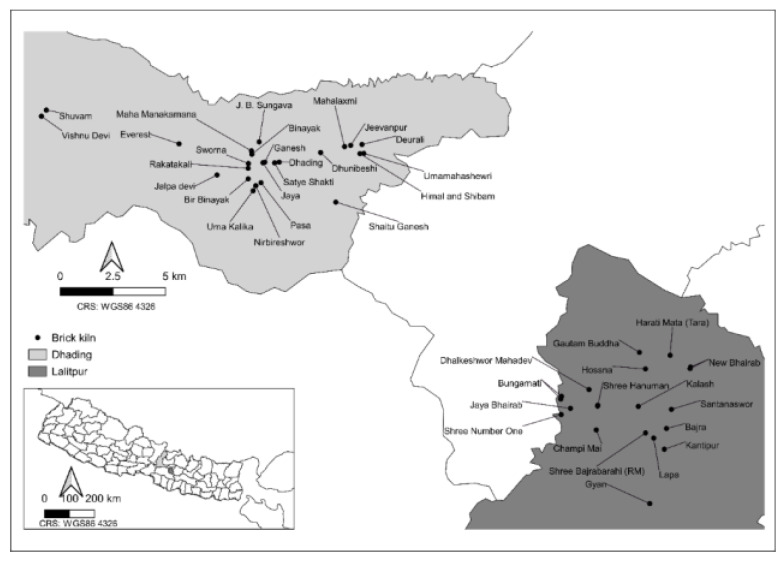
Location of the Nepalese brick kilns where equid welfare assessments took place between December 2018 and April 2019. Kilns were located in two districts of Nepal separated by Kathmandu. Black dots represent the locations of the brick kilns in Dhading and Lalitpur. The inset map shows all the regions of Nepal, with Dhading and Lalitpur shaded in their respective colours.

Specific EARS surveys relevant to brick kilns were developed which were comprised of questions relating to equid welfare [23]. The EARS tool is a questionnaire-based method of collecting welfare assessment data in a standardised and stratified way. The EARS tool is designed to obtain individual information about an equid and its surrounding environment, or about a group of equids for analysis to take place at the population level. EARS assessments collect standardised information relating to equid demography, living conditions, working conditions, health and nutrition. EARS assessment forms were collected on mobile devices (tablet/smartphone) using the ODK Collect app [23,28] by trained enumerators from Animal Nepal (a local animal welfare NGO) between December 2018 and April 2019 at 41 brick kilns (Figure 1). All participants gave their informed consent for inclusion before they participated in this study. This study was conducted in accordance with the Declaration of Helsinki, and the protocol was approved by the Ethics Committee of The Donkey Sanctuary UK under Project Number 2019-AIM2-NEPAL.

All statistical analyses were performed using R v5.2 [28] and RStudio v1.2 [29]. R package ‘dplyr’ was used to count the number of observations for each question and tally the responses [30]. Differences in the number of equids within each brick kiln and each region, as well as each species and sex category were tested using type III analysis of variance using the R package ‘stats’ [28]. Where responses to questions were not normally distributed, Box-Cox transformations were applied to ensure data conformed to normality assumptions [31]. Type III analysis of variance was used to account for the unbalanced experimental design using the R package ‘car’ [32]. Further Tukey honest significance test (HSD) post-hoc tests were used to understand within-group variation, for which the analysis of variance test also included parameters to account for the unbalanced project design and adjust *p*-values accordingly using the R package ‘agricolae’ [33]. 

The WAG tool was used to summarise the welfare conditions of equids in brick kilns following the same methodology as Kubasiewicz et al. [15]. In brief, the WAG tool uses key indicator questions from EARS relating to nutrition, health, behavior, working and housing conditions to assign welfare grades for each of these categories for a population of equids. The first step assigns category scores to individuals based on good (score of 25), medium (score of 12.5) or bad (score of 1) welfare using decision trees. The second step sums the scores of each question into an overall grade per category for the equid. The third step combines the grade for a population of equids where the overall grade is assigned as the lowest grade for a cumulative total of at least 15% of the animals in the group, where percentages are accumulated from the worst grade ‘J’ (1 to 10) to the best ‘A’ (90 to 100). WAG grades for each category level within the following indicators: species, age, sex, work type and location (district) were used to highlight areas of welfare concern. Each WAG category consists of four main questions, with alternate questions in some cases where it might not be possible to answer a main question (e.g., those requiring the presence of an owner/handler). In this study, the main WAG questions ‘For how long is fibre available’ was replaced throughout with the alternate question ‘Presence of a clean water point whilst resting’ due to a lack of data, where a water point is defined as any area in which water is provided to equids. The question ‘Age the equid started working’ was replaced with ‘Access to shade during work breaks’ due to lack of data.

Where welfare concerns were identified using the WAG tool, questions contained within each WAG category were investigated using analysis of variance with the R package ‘stats’ [28] using the same procedure as described above. 

To understand if working with the handler could improve the welfare of equids in Nepalese brick kilns, categorical descriptions were used to describe the handler’s behaviour in relation to the health, behaviour, nutrition, housing and working conditions of those equids [34]. Categorical descriptions evaluate the links between the responses to EARS questions, in this case handlers behaviour in relation to the equid, and the other responses to the EARS question (global) of interest by comparing two proportions [34,35]. 

## 3. Results

In total, 2,448 equids at 41 brick kilns were assessed using the EARS tool; 55 (2%) were donkeys, 1028 (42%) were mules and 1365 (56%) were horses. The equids were worked by 126 different handlers at 41 different brick kilns. In Lalitpur, we surveyed 68 different handlers across 17 different brick kilns. In Dhading, we surveyed 58 different handlers across 24 different brick kilns (Figure 1). 

### 3.1. Demography

**Figure 2:**
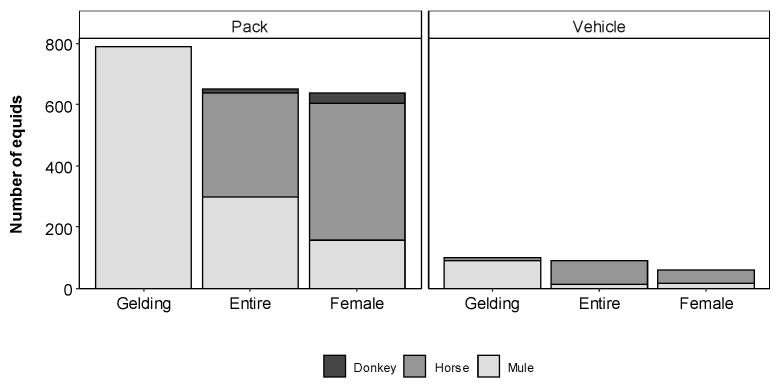
Number of equids by species and sex surveyed in the brick kilns, differentiated by brick transportation method used.

**Figure 3:**
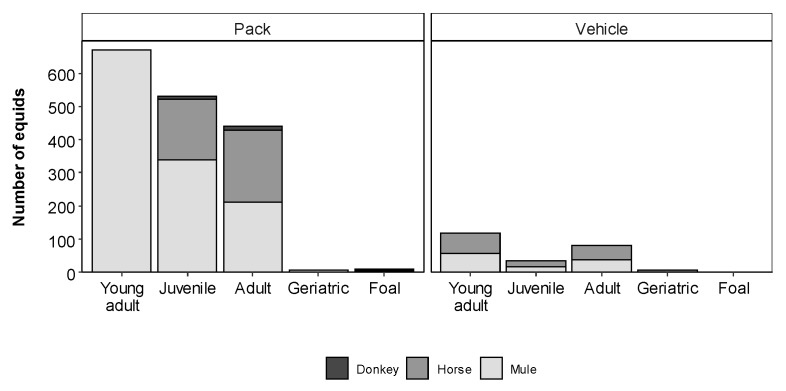
Number of equids by species and age category surveyed in the brick kilns, differentiated by the brick transportation method used.

There was a large amount of variation in the number of equids working at the different kilns (min = 8, max = 64, mean = 33, s.d = 12.8, *p <* 0.001). Despite Champi Mai in Lalitpur having the largest population of working equids (*n* = 64), Dhading had kilns with significantly larger populations of working equids overall (P-adjusted 0.003). Equids were most frequently castrated males, more commonly referred to as geldings, followed by stallions, with the least frequent gender of equid being female. More equids transported bricks using packsaddles than with vehicles (Figure 2). There were significantly more young adult equids compared with adults, juveniles, geriatrics and foals (*p*-adjusted < 0.001, Figure 3). 

### 3.2. Welfare Aggregation

All demographics obtained a nutrition grade of G, except geriatric equids, which obtained a grade of E, indicating a generally poor nutritional status of equids working in brick kilns independent of district, species, sex or type of work. The health of horses and mules obtained a WAG grade C; however, donkey health was graded more poorly at WAG grade D (Table 1). 

Behaviour achieved an overall WAG grade of D but differed between district, sex, species and type of work. Equids working in Dhading had better behaviour grades compared with those in Lalitpur. Males had a lower behavioural WAG grade than females. Donkeys and mules had a lower behaviour grade compared to horses. Where equids were using packs to transport bricks, their WAG behaviour grade was better than those that transported bricks via a vehicle (Table 1).

Housing varied between groups but overall achieved a WAG grade of D, suggesting scope for improvement. Housing conditions varied depending on work type, with equids using vehicles to transport bricks achieved a housing grade of G, compared to a B for pack animals. Housing in Dhading rather than Lalitpur, and that of donkeys and mules rather than horses, was generally found to be better, with the former groups achieving a grade of B (Table 1) 

The overall working WAG grade was H. However, equids that transported bricks by vehicle, as well as young adults and donkeys were graded at G, indicating better working conditions for young adults and donkey (Table 1). 

The drivers of these poor levels of welfare may differ between WAG indicators, we will explore these further for different species to better understand welfare conditions in the study kilns.

### 3.3. Nutrition 

The nutrition WAG covers four EARS questions relating to body condition, feed, water quality and availability. The questions with the highest proportion of equids with poor welfare in the nutrition category were 'access to clean water while working’ (95.7% limited access) and ‘presence of a clean water point while housed’ (97.7% limited access), both of which had significantly more responses than ‘no access’ or ’free access’ to water (Table 2). Water was frequently given by handlers at distinct intervals in small buckets rather than being available at an accessible water source, such as a trough, therefore providing only limited access was driving the poor WAG grade for nutrition, and signifies an area for improvement. 

Overall, there were significantly more equids with thin/moderate and ideal body condition scores compared with those that were very thin or poor, fat or very fat. The majority of donkeys and horses were found to have a thin/moderate body condition score; however, greater numbers of mules had an ideal body condition (Table 2) suggesting that mules may be better able to maintain an ideal body condition when facing the same conditions as horses or donkeys.

There were 25 different types of feed reported as fed to the equids in this study, the most frequent of which are presented in Table 2. Pasture access was most frequently provided to equids (33%), whilst 17% of equids were fed straights with vitamins and minerals, while cereal grains and muesli were fed to ~12% of the equids. Straw, chopped fibre, hay and haylage were only fed to a small proportion of equids assessed (Table 2). Straights are whole or crushed grains such as oats or corn, not nutritionally complete if given on their own but are used to supplement diet, particularly equids being worked.

### 3.4. Health

More equids obtained poor welfare scores for ‘signs of skin alterations’ than any other question within the health category, driving the poor WAG grade for health overall. There were significantly more equids with scars compared to any other skin alteration and significantly more equids had alopecia compared with sarcoids or swellings. However, only a small proportion of equids with sarcoids or swellings were encountered during the EARS assessments (Table 3). High prevalence of scars, alopecia and open wounds lowered the WAG score. 

A majority of the equids had a healthy coat and few equids were found to be lame. Only 0.5% were severely lame and unable to move. Despite few equids being found to be lame overall, significantly more donkeys were severely lame compared to mules and horses (Table 3).

There were few equids that showed obvious signs of illness, however 8.4% of the total population did show signs of eye discharge and 6.1% showed signs of nasal discharge.

### 3.5. Behaviour

More equids showed signs of harmful practices than any other indicator of poor welfare in the behaviour category. Significantly more equids showed signs of tethering and hobbling than signs of firing, amputation or mutilation, wounds from poor fitting harnesses or the use of rope nosebands made from abrasive material, in direct contact with the skin (Table 4). Signs of tethering include old scarring indicated by white hairs, bare skin, abraded hair and open wounds. The presence of these harmful practices observed at the kilns was the main driver of the poor behaviour WAG grade, suggesting that it was the behaviour of the handler towards the equid that was reducing welfare rather than the behaviour of the equid. 

For each equid, their general attitude at a distance was assessed to understand their emotional state. Significantly more equids were found to be at ease compared with other attitudes expressed.

Equids were also assessed for signs of fear, with most of the equids showing no signs. For those showing signs of fear, the most frequent were unpredictable or sudden movements, showing the whites of their eyes and displaying head shyness (Table 4).

Significantly more handlers were cautious or fearful as opposed to assertive or indifferent, or aggressive towards their equids. When considering each species, the percentage of cautious/fearful handlers was higher than average for mule handlers, and lower for donkey handlers, whilst 8.6% of donkey handlers were assertive/indifferent (Table 4).

### 3.6. Working Conditions

The working condition WAG grade includes the number of days worked per week, hours worked per day, if equids had an opportunity for rest and if shade was provided during rest periods. A majority of equids worked 6 days per week for 6 to 9 h per day. Although the equids did have an opportunity for rest during the day, their equipment was not removed, and they had limited access to shade (Table 5) and water (Table 2). Generally, all the WAG component questions had a similar bearing on the low working grade indicating that there are several areas for improvements in working practices to improve the welfare of equids.

### 3.7. Housing Conditions

When not working, the majority of equids were kept indoors, which contributed the highest proportion of poor welfare responses, and was the main driver of the poor WAG grade for housing. A similar proportion of equids were found in environments that were not clean and free from hazards, did not have satisfactory dimensions and lacked a clean, dry resting area. All three of these welfare issues were more prevalent for donkeys than mules or horses (Table 6).

### 3.8. Handlers Attitudes towards Equids

Relaxed and confident handlers had more equids with no signs of hoof neglect or disease (95% vs. 94% global, *p <* 0.001), however, body condition was generally moderate (39% vs. 38% global, *p =* 0.001). The general attitude of equids handled by relaxed and confident handle was at ease (85% vs. 82% global, *p <* 0.001), and the equids were friendly towards the enumerator (17.5% vs. 16.6% global, *p <* 0.001) showing no signs of fear or distress (22% vs. 21% global, *p <* 0.001). However, their equids were provided partially dirty water (20% vs. 19% global, *p =* 0.014) but access was unlimited (70% vs. 71% global, *p =* 0.008). Relaxed and confident handlers were more likely to provide shelters of sufficient size (79% vs. 78% global, *p <* 0.001), with sufficient bedding material (1.4% vs. 1.3% global, *p <* 0.001).

Cautious and fearful handlers had equids in ideal body condition (68% vs. 55% global, *p =* 0.003) with no signs of skin alterations (63% vs. 48% global, *p =* 0.002). However, their equids generally either moved their heads away (7% vs. 2%, *p <* 0.001) or were aggressive (2% vs. 0.6% global, *p =* 0.002) towards the enumerator. Despite this, they did have the opportunity for social interaction with other animals (39% vs. 20% global, *p <* 0.001). In contrast to relaxed and confident handlers, during rest breaks, cautious and fearful handlers provided clean water (92% vs. 80% global, *p =* 0.022) but access was limited (86% vs. 71% global, *p =* 0.015) and the stable bedding was insufficient (2% vs. 0.8% global, *p =* 0.001).

Assertive handlers generally had equids that were in ideal body condition (100% vs. 55% global, *p =* 0.005) but the equids were often lame (100% vs. 5% global, *p* < 0.001), had swellings (33% vs. 1% global, *p <* 0.001) and open wounds (33% vs. 9%, *p =* 0.045). Their equids also had no opportunity for social interaction with other animals (100% vs. 34% global, *p <* 0.001) and the handlers had no direct interaction with their equids at the time of the EARS assessment (100% vs. 52 global, *p <* 0.001). Assertive handlers typically provided housing insufficient in size (100% vs. 21.3%, *p <* 0.001) with insufficient bedding (34% vs. 21% global, *p <* 0.001). 

Equids with aggressive handlers generally moved their entire body away from the observer (16% vs. 0.5% global, *p <* 0.001) and showed signs of aggressive behaviour (20% vs. 0.5% global, *p <* 0.001), being agitated, hyper-reactive and hyper-vigilant (17% vs. 1% global, *p <* 0.001) and showing the whites of their eyes (8% vs. 1.2% global, *p =* 0.040). Aggressive handlers often used rough physical interactions such as slapping, hitting and yanking (17% vs. 0.2% global, *p <* 0.001) and rested against the equids while the assessment was taking place (33% vs. 3% global, *p <* 0.001).

## 4. Discussion

Overall, equids working in Nepalese brick kilns were found to mostly be young adult gelded mules (Figure 2). This is a similar demography to equids working in Egyptian brick kilns [16]. The majority of equids had an ideal or moderate body condition score which is consistent with other findings by Ali et al. [16] in Egypt but contrasts from Pakistan [12] where body condition was found to be thin and attributed to poor nutrition. Few equids in this study showed signs of lameness [36]. 

Scarring was the most frequent skin alteration across all three species working in the kilns, there was also a high proportion of hobbling and tethering. Issues relating to scars and tethering become worse over time especially when an equid is working constantly for an entire brick kiln season [26]. Such anthropogenic pressures on equids can have detrimental effects on their welfare [5,11,16]. Limb tethering or hobbling are often used in LMICs to restrict the mobility of equids and is likely to be the cause of the scarring observed on many equids [3,26]. Common reasons can be the repeated use of inappropriate coarse materials combined with accumulation of substrate matter which abrades the skin [24]. These findings highlight that harmful practices have a detrimental impact on the welfare of working equids, especially those working for long periods with inadequate opportunities to rest [2,37]. 

In terms of behaviour, many equids assessed were found to be at ease, calm and relaxed. Additionally, most equids showed no signs of fear or distress. Overall, this indicates that a typical equid within a Nepalese brick kiln is not in a general state of agitation when at rest or during work. However, this cannot be immediately attributed to a lack of fear or distress in the equids; it may be a behavioral manifestation of learned helplessness or hopelessness [38]. Our results suggest there was a general trend whereby when equid handlers are aggressive towards their equids, the equids display aggressive behaviour and fear responses such as showing the whites of their eyes and moving away from observers. Combinations of poor harnessing and inappropriate nosebands, overwork and inhumane handling lead to a vicious cycle of escalating conflict behaviours, which in turn escalate human responses. Resulting physiological stress can impair immune responses and hinder tissue repair [39]. These findings are similar to other studies which found that when equids are treated in an aggressive manner, they are more likely to suffer from mistreatment [16] and poorer welfare standards [3,12,16,26]. In contrast, handlers that were relaxed with their equids had equids that were at ease, friendly to the approach of the enumerator, had visual contact with other animals and showed no signs of fear. These equids were also found to have access to clean water when working and housing of satisfactory size with clean bedding, indicating that the positive attitude of handlers towards their equids was promoting better welfare and more relaxed behaviour in their equids and is in concurrence with other studies [3,12,16,26,40].

Despite eye and nasal discharge being the most frequent sign of illness, the working environment was found to be generally clean and free from hazards. Equids routinely worked between six and nine hours a day and for six days per week. These outcomes differ from the study by Ali et al. [16] where mules working in Egyptian brick kilns typically worked longer but those equids were found to have comparatively poorer welfare. In the Nepalese brick kilns, when resting, equids frequently had limited access to shade and water. Equids also did not have their equipment removed, which may have been a contributing factor towards the large proportion of scars observed. To improve the working conditions of these equids their equipment should be removed during the rest periods and they should be given unlimited access to clean water, appropriate feed and shade. However, this would require a change in working practices as suggested by other studies [24,41].

Outside of the working period, the majority of equids had limited access to partially dirty water. Most equids were stabled during these rest periods with only a quarter having access to grazing during these times. Grazing, although probably limited in quality and quantity in the brick kiln environment, is known to be beneficial to welfare and could provide some relief particularly where stable size is insufficient. Donkeys, in particular, were kept in housing of insufficient size with inadequate or soiled bedding, potentially depriving animals of adequate rest between work periods. Donkeys are typically owned by the poorest people so are commonly subjected to lower quality husbandry through lack of education and economic pressures [37]. Equids in housing preventing sufficient periods of recumbence may suffer reduced lying behaviour and other negative health responses [42,43,44,45], these debilitated animals may then be subjected to increasingly harsh handling in order to maintain productivity [46]. Equipment removal and access to shade and clean water would allow animals to feed, rehydrate, stretch and sleep when not working. This is particularly important as brick kilns are harsh environments for equids to live and work in [12], with high temperatures and solar radiation levels posing welfare concerns that can ultimately lead to heat stress [9].

Based on the results from EARS assessments and WAG grading of equid welfare conditions in the brick kilns of Nepal, improvements should focus on a better provision of shade, clean water and adequate feed as well as removing equipment during rest breaks. Currently equipment is not removed during breaks and equid access to water and shade is limited. If equipment were to be removed and free access to shade and clean water given, this would allow animals to rehydrate, cool down, stretch or lie down and allow wounds to have respite from continuous interference during the working day. In addition, this study highlights that there should be improvements to the housing conditions of equids by ensuring adequate space provision, sufficient non-soiled bedding material and free access to clean water, as these would benefit the welfare of equids working in these brick kilns. 

The WAG tool highlights that the health of the equids working in brick kilns was generally comparable to results found in larger scale studies [15], however, the behaviour grades were generally lower [15]. Further investigation of the poor behaviour grades revealed that a trend for both the use of harmful practices and negative attitudes of the handlers towards the equids was driving a lower WAG grade than observed in other studies [15]. These results suggest that further work to improve the health and behaviour of equids in these kilns should focus on training the handlers in appropriate methods for handling their equids and improving working practices to reduce both injuries and harmful practices [47]. Effective methods for communicating and implementing good equid welfare practices including contact with specifically trained animal health workers applied in combination with demonstrations, presentations and visual aids such as pamphlets and posters could benefit the welfare of equids working in brick kilns [48]. 

## 5. Conclusions

Applying the WAG tool to the responses of EARS questions as part of an equid welfare framework allowed areas of welfare concern to be quickly highlighted and appropriate intervention measures to be proposed, which if adopted, could improve the general welfare of equids working in brick kilns. 

Specific to the Nepalese brick kilns, further work to improve the behaviour, housing, health, nutrition and working conditions and mechanisms for achieving this have been discussed. The welfare concerns highlighted by this study are anthropogenic, and as such, could conceivably be overcome by culturally sensitive, bespoke educational programs and training. For example, training relating to the importance of removing packs during rest periods could help reduce injuries associated with using packs to carry bricks. Interventions regarding handling and equid management should be aimed at both equid handlers, equid owners and brick kiln owners and delivered by trained professionals and paraprofessionals as this would ensure a strong, consistent message is developed at all levels. Further education and training could also target the importance of providing clean, dry sufficient bedding and the importance of not limiting access to water or shade and providing good quality feed. 

Any interventions should be subject to a rigorous monitoring and evaluation system to ensure objectives are achieved. The EARS and WAG tools, in combination, provide an appropriate equid welfare monitoring framework within which to achieve these goals. Using the EARS framework and WAG grades gives a rapid and transparent methodology to indicate how future resources can be best directed to improve the welfare of equids in Nepalese brick kilns and is transferable to a range of other situations. In addition to providing clear insights, a solid basis has also been established to enable comparison with other brick kilns around the world. Future work should focus on two aspects, firstly, monitoring change over time in these established brick kilns and, secondly, applying this methodology to brick kilns in other countries to understand equid welfare under different environmental and socio-economic contexts.

## Figures and Tables

**Table 1:** Welfare Aggregation Grades obtained for each category of population: district, sex, species, age and type of work. Foals were excluded from the analysis due to lack of data.

	Nutrition	Health	Behaviour	Housing	Working
**Overall WAG Category**	G	C	D	D	H
**District**					
Dhading	G	C	C	B	H
Lalitpur	G	C	E	D	H
**Sex**					
Female	G	C	C	D	H
Gelding	G	C	D	D	H
Jack/stallion/entire	G	C	D	D	H
**Species**					
Donkey	G	D	E	B	G
Horse	G	C	C	D	H
Mule	G	C	D	B	H
**Age Category**					
1 to 3 years (Juvenile)	G	C	C	D	H
3 to 5 years (Young adult)	G	C	D	D	G
>5 years (Adult)	G	C	E	D	H
>20 years (Geriatric)	E	C	C	B	H
**Brick Transportation**					
Pack	G	C	D	B	H
Vehicle	G	C	E	G	G

**Table 2:** Nutrition parameters that were assessed at the 41 brick kilns in Nepal. The total number of equid assessments was *n* = 2448. Donkey, mule and horse nutritional conditions are expressed as a total of the species population within each category. Tukey-HSD comparisons of results within each question are marked with different letters when there were significantly different proportions of equids within each response.

Welfare Level	Response	% within Each Species			Overall (%)
		Donkey	Horse	Mule	
**Body Condition Score**					
Bad	Very thin/poor	1.9 ^c^	9.1 ^c^	2.1 ^c^	4.9 ^b^
Medium	Thin/moderate	58.5 ^a^	50.0 ^a^	42.3 ^a^	46.1 ^a^
Good	Ideal	37.7 ^b^	38.8 ^b^	53.4 ^b^	46.4 ^a^
Medium	Fat	1.9 ^c^	2.2 ^c^	2.2 ^c^	2.0 ^b^
Bad	Very fat	0.0	0.0	0.0	0.0
**Is the equid getting an appropriate diet?**					
Good	Pasture	25.8 ^abc^	27.4 ^bc^	27.4 ^abc^	32.7 ^d^
Good	Vitamins/minerals	8.3 ^abc^	10.9 ^abc^	10.9 ^abc^	12.9 ^cd^
Good	Browse	6.9 ^abc^	3.5 ^abc^	2.6 ^abc^	3.7 ^cd^
Good	Straw/stover	0.5 ^c^	0.6 ^ab^	0.9 ^ab^	0.9 ^ab^
Good	Chopped fibre/chaff	0.0	1.1 ^abc^	0.7 ^ab^	1 ^abcd^
Good	Hay	0.0	0.4 ^abc^	0.6 ^abc^	0.6 ^abc^
Good	Haylage	0.0	0.1 ^a^	0.2 ^a^	0.2 ^a^
Medium	Straights	16.1 ^abc^	15.1 ^bc^	14.4 ^abc^	17.6 ^d^
Medium	Creep feed	5.5 ^abc^	2.8 ^abc^	2.8 ^abc^	3.4b ^cd^
Medium	Legumes/pulses	3.2 ^abc^	1.9 ^abc^	1.2 ^abc^	1.8 ^d^
Bad	Cereal grains	10.6 ^abc^	9.9 ^abc^	10.8 ^abc^	12.5 ^cd^
Bad	Mix/muesli	5.5 ^abc^	9.8 ^abc^	10.8 ^abc^	12.2 ^cd^
Bad	Silage	0.0	0.2 ^abc^	0.5 ^abc^	0.4 ^abc^
**Access to clean water during working period**					
Good	Free access	0.0	2.7 ^bc^	4.5 ^b^	3.9 ^b^
Medium	Limited access	95.7 ^a^	96.5 ^a^	94.0 ^a^	95.7 ^a^
Bad	No access	4.3 ^b^	0.8 ^c^	1.5 ^c^	0.4 ^b^
**Presence of a clean water point (housed)**					
Good	Free access	0.0	2.2 ^b^	1.6 ^b^	1.8 ^b^
Medium	Limited access	96.1 ^a^	97.3 ^a^	98.3 ^a^	97.7 ^a^
Bad	No access	3.9 ^b^	0.5 ^c^	0.1 ^c^	0.5 ^b^

**Table 3:** Health parameters that were assessed at the 41 brick kilns in Nepal. The total number of equid assessments was *n* = 2448. Donkey, mule and horse health conditions are expressed as a total of the species population within each category. Tukey-HSD comparisons of results within each question are marked with different letters when there were significantly different proportions of equids within each response.

Welfare Level	Response	% within Each Species			Overall (%)
		Donkey	Horse	Mule	
**Signs of lameness**					
Good	No lameness	90.7 ^a^	93.8 ^a^	93.6 ^a^	93.7 ^a^
Medium	Lame but still moving	5.6 ^b^	5.7 ^b^	6.0 ^b^	5.8 ^b^
Bad	Severely lame	3.7 ^b^	0.5 ^c^	0.4 ^c^	0.5 ^c^
**Is the equid’s coat healthy?**					
Good	Yes	91.5 ^a^	88.8 ^a^	92.4 ^a^	90.8 ^a^
Bad	No	8.5 ^b^	11.2 ^b^	7.6 ^b^	9.2 ^b^
**Skin alterations**					
Good	No signs present	40.3 ^a^	41.4 ^a^	36.1 ^a^	37.9 ^a^
Medium	Scars	19.4 ^b^	27.9 ^ab^	33.7 ^a^	30.9 ^a^
Medium	Alopecia	18.1 ^b^	16.8 ^b^	21.2 ^ab^	19.4 ^b^
Medium	Swellings	4.2 ^c^	1.3 ^cd^	1.2 ^cd^	1.5 ^c^
Bad	Open wounds	18.1 ^b^	12 ^bc^	7.4 ^c^	9.7 ^bc^
Bad	Sarcoids	0.0	0.7 ^d^	0.4 ^d^	0.5 ^c^
**Obvious signs of illness**					
Good	No signs present	61.1 ^a^	80.3 ^a^	88.4 ^a^	84.2 ^a^
Bad	Eye discharge	11.1 ^b^	11.4 ^b^	5.9 ^c^	8.4 ^b^
Bad	Nasal discharge	19.4 ^b^	6.9 ^c^	4.7 ^c^	6.1 ^b^
Bad	Signs of diarrhoea	8.3b ^c^	0.5 ^d^	0.3 ^d^	0.6 ^c^
Bad	Discharge (penis or vulva)	0.0	0.2 ^d^	0.3 ^d^	0.2 ^c^
Bad	Abdominal pain	0.0	0.7 ^d^	0.4 ^d^	0.5 ^c^

**Table 4:** Behaviour of the equids and owner behaviour towards their equids at 41 brick kilns in Nepal. The total number of equid assessments was *n* = 2448; responses are expressed as a proportion of the total population within each category for each species. Tukey-HSD comparisons of results within each question are marked with different letters when there were significantly different proportions of equids within each response.

Welfare Level	Response	% within Each Species			Over All (%)
		Donkey	Horse	Mule	
**Harmful practices**					
Good	No	61.4 ^a^	60.0 ^a^	56.3 ^a^	58.1 ^a^
Bad	Limb tethering or hobbling	31.6 ^b^	37.6 ^b^	42.5 ^b^	41.1 ^b^
Bad	Amputations or mutilations	3.5 ^c^	0.1 ^c^	0.1 ^c^	0.2 ^c^
Bad	Firing and hot branding	3.5 ^c^	0.2 ^c^	0.2 ^c^	0.3 ^c^
Bad	Rope noseband	0.0	0.0	0.1 ^c^	0.1 ^c^
**General attitude of the equid at a distance**					
Good	At ease	83.6 ^a^	76.6 ^a^	70.1 ^a^	72.8 ^a^
Good	Alert and active	14.5 ^b^	21.1 ^b^	26.5 ^b^	24.1 ^b^
Bad	Agitated, aggressive	0.0	1.1 ^c^	1.1 ^c^	1.1 ^c^
Bad	Apathetic, depressed, withdrawn	1.8 ^c^	1.3 ^c^	2.3 ^c^	1.9 ^c^
**Signs of fear and distress**					
Good	No signs of fear and distress present	75.0 ^a^	77.7 ^a^	72.9 ^a^	75.0 ^a^
Bad	Head shyness	10.0 ^b^	5.2 ^bc^	3.8 ^bc^	4.5 ^bc^
Bad	Unpredictable or sudden movements	8.3 ^b^	8.2 ^b^	9.1 ^b^	8.7 ^b^
Bad	Showing the whites of the eyes	0.0	5.1 ^bc^	9.3 ^b^	7.1 ^b^
Bad	Sudden startle responses	3.3 ^bc^	1.4 ^c^	1.2 ^c^	1.4 ^c^
Bad	Aggressive behaviour	1.7 ^c^	2.1 ^bc^	3.2 ^bc^	2.8 ^cd^
Bad	Trembling	1.7 ^c^	0.4 ^cd^	0.3 ^cd^	0.3 ^d^
**Handlers interaction when holding the equid**					
Good	Relaxed and confident	91.4 ^a^	92.8 ^a^	90.4 ^a^	91.4 ^a^
Medium	Cautious/fearful	0	6.9 ^b^	9.0 ^b^	7.9 ^b^
Medium	Assertive/indifferent	8.6 ^b^	0	0	0.2 ^c^
Bad	Aggressive	0	0.4 ^c^	0.6 ^c^	0.5 ^c^

**Table 5:** Equid working conditions that were assessed at the 41 brick kilns in Nepal. The total number of equid assessments was *n* = 2448. Donkey, mule and horse health conditions are expressed as a total of the species population within each category. Tukey-HSD comparisons of results within each question are marked with different letters when there were significantly different proportions of equids within each response.

Welfare Level	Response	% within Each Species			Over All (%)
		Donkey	Horse	Mule	
**Number of working hours per day**					
Good	More than 3, less than or equal to 6 h	8.8 ^b^	5.2 ^b^	5.5 ^b^	5.4 ^b^
Good	Less than or equal to 3 h	1.8 ^c^	0.1 ^d^	0	0.8 ^cd^
Medium	More than 6, less than or equal to 9 h	86.0 ^a^	94.2 ^a^	93.5 ^a^	93.6 ^a^
Bad	More than 9 h	1.8 ^c^	0.6 ^d^	0.9 ^d^	0.1 ^d^
**Number of days worked per week**					
Good	5 days or less	1.8 ^c^	1.2 ^c^	0.7 ^c^	1.0 ^c^
Medium	6 days	91.1 ^a^	84.6 ^a^	86.4 ^a^	85.7 ^a^
Bad	7 days	7.1 ^bc^	14.2 ^b^	12.9 ^b^	13.3 ^b^
**Does the equid have a rest break during the working day**					
Good	yes, equipment removed	1.8 ^c^	13.3 ^b^	7.3 ^bc^	9.6 ^b^
Medium	yes, equipment not removed	76.4 ^a^	72.5 ^a^	84.8 ^a^	79.1 ^a^
Bad	No	21.8 ^b^	14.2 ^b^	7.9 ^bc^	11.2 ^b^
**Does the equid have access to shade during breaks**					
Good	Access to shade	37.2 ^b^	23.8 ^b^	23.4 ^b^	23.8 ^b^
Medium	Limited access to shade	58.1 ^ab^	72.4 ^a^	73.6 ^a^	72.8 ^a^
Bad	No access to shade	4.7 ^c^	3.7 ^c^	2.9 ^c^	3.3 ^c^

**Table 6:** Housing parameters that were assessed at the 41 brick kilns in Nepal. The total number of equid assessments was *n* = 2448. Donkey, mule and horse housing conditions are expressed as a total of the species population within each category. Tukey-HSD comparisons of results within each question are marked with different letters when there were significantly different proportions of equids within each response.

Welfare Level	Response	% within Each Species			Overall (%)
		Donkey	Horse	Mule	
**Housing regime**					
Good	Stabled equid—access to field	30.5 ^b^	26 ^b^	24.6 ^b^	25.3 ^b^
Good	Kept outside—access to shelter	0.0	0.8 ^c^	0.1 ^c^	0.4 ^c^
Medium	Indoor housing	69.5 ^a^	73.1 ^a^	75.0 ^a^	74.1 ^a^
Bad	Kept outside—no access to shelter	0.0	0.0	0.0	0.0
**Is the environment clean and free from hazards**					
Good	Yes	65.5 ^a^	74.9 ^a^	85.3 ^a^	72.7 ^a^
Bad	No	34.5 ^b^	25.1 ^b^	14.7 ^b^	27.2 ^b^
**Dimensions of the stable/shelter?**					
Good	Satisfactory	61.0 ^b^	69.8 ^ab^	85.4 ^a^	78.2 ^a^
Bad	Not-Satisfactory	39.0 ^c^	30.2 ^c^	14.6 ^c^	21.7 ^b^
**Is there a clean, dry comfortable lying area**					
Good	Yes	44.1 ^b^	70.7 ^a^	82.7 ^a^	76.7 ^a^
Bad	No	55.9 ^b^	29.3 ^c^	17.3 ^c^	23.2 ^b^
